# Routes of Drug Use Among Drug Overdose Deaths — United States, 2020–2022

**DOI:** 10.15585/mmwr.mm7306a2

**Published:** 2024-02-15

**Authors:** Lauren J. Tanz, R. Matt Gladden, Amanda T. Dinwiddie, Kimberly D. Miller, Dita Broz, Eliot Spector, Julie O’Donnell

**Affiliations:** ^1^Division of Overdose Prevention, National Center for Injury Prevention and Control, CDC; ^2^Division of HIV Prevention, National Center for HIV, Viral Hepatitis, STD, and TB Prevention, CDC; ^3^Oak Ridge Institute for Science and Education, Oak Ridge, Tennessee.

**Summary**

**What is already known about this topic?**

More than 109,000 drug overdose deaths occurred in the United States in 2022; nearly 70% involved illegally manufactured fentanyls (IMFs). Data from the western United States suggested a transition from injecting heroin to smoking IMFs.

**What is added by this report?**

From January–June 2020 to July–December 2022, the percentage of overdose deaths with evidence of smoking increased 73.7%, and the percentage with evidence of injection decreased 29.1%; similar changes were observed in all U.S. regions. Changes were most pronounced in deaths with IMFs detected, with or without stimulant detection.

**What are the implications for public health practice?**

Strengthening and expanding public health and harm reduction services to address overdose risk with smoking and other noninjection routes might reduce deaths.

## Abstract

Preliminary reports indicate that more than 109,000 drug overdose deaths occurred in the United States in 2022; nearly 70% of these involved synthetic opioids other than methadone, primarily illegally manufactured fentanyl and fentanyl analogs (IMFs). Data from the western United States suggested a transition from injecting heroin to smoking IMFs. CDC analyzed data from the State Unintentional Drug Overdose Reporting System to describe trends in routes of drug use in 27 states and the District of Columbia among overdose deaths that occurred during January 2020–December 2022, overall and by region and drugs detected. From January–June 2020 to July–December 2022, the percentage of overdose deaths with evidence of injection decreased 29.1%, from 22.7% to 16.1%, whereas the percentage with evidence of smoking increased 73.7%, from 13.3% to 23.1%. The number of deaths with evidence of smoking increased 109.1%, from 2,794 to 5,843, and by 2022, smoking was the most commonly documented route of use in overdose deaths. Trends were similar in all U.S. regions. Among deaths with only IMFs detected, the percentage with evidence of injection decreased 41.6%, from 20.9% during January–June 2020 to 12.2% during July–December 2022, whereas the percentage with evidence of smoking increased 78.9%, from 10.9% to 19.5%. Similar trends were observed among deaths with both IMFs and stimulants detected. Strengthening public health and harm reduction services to address overdose risk related to diverse routes of drug use, including smoking and other noninjection routes, might reduce drug overdose deaths.

## Introduction

Preliminary data indicate that U.S. drug overdose deaths surpassed 109,000 in 2022; nearly 70% of these deaths involved synthetic opioids other than methadone, primarily illegally manufactured fentanyl and fentanyl analogs (IMFs).* In recent years, deaths co-involving IMFs and stimulants have increased steadily (*1*). The estimated number of U.S. adults who inject drugs increased from approximately 774,000 in 2011 to nearly 3.7 million in 2018, corresponding to shifts from prescription opioid misuse to the use of heroin and IMFs (*2*). More recent data suggest transitions from injecting heroin to smoking IMFs; however, limited data exist on recent changes in routes of drug use for all drugs, and for IMFs beyond the western United States^†^ (*3**,**4*). Routes of drug use have implications for overdose risk, infectious disease transmission, other comorbidities, and harm reduction services (*5*).

## Methods

Jurisdictions entered data from death certificates, postmortem toxicology testing, and medical examiner or coroner reports on unintentional and undetermined intent drug overdose deaths into CDC’s State Unintentional Drug Overdose Reporting System (SUDORS).^§^ Routes of drug use were identified using information from scene investigations, witness reports, or autopsy data and were categorized into nonmutually exclusive categories of ingestion,^¶^ injection,** smoking,^††^ and snorting^§§^; other routes (e.g., transdermal) are not presented because sample sizes were small. Among 28 jurisdictions^¶¶^ with complete data,*** numbers and percentages of overdose deaths were calculated by route of drug use and by 6-month period during January 2020–December 2022, overall, and for each U.S. Census Bureau region.^†††^ To understand how routes of drug use are related to drugs commonly involved in overdose deaths, percentages of overdose deaths with evidence of each route were calculated by 6-month period for mutually exclusive categories of drugs detected (IMFs^§§§^ only, stimulants only, both IMFs and stimulants, and neither IMFs nor stimulants)^¶¶¶^ (*6*). Analyses were performed using SAS software (version 9.4; SAS Institute). This activity was reviewed by CDC, deemed not research, and was conducted consistent with applicable federal law and CDC policy.****

## Results

### Overall Trends

**FIGURE 1:**
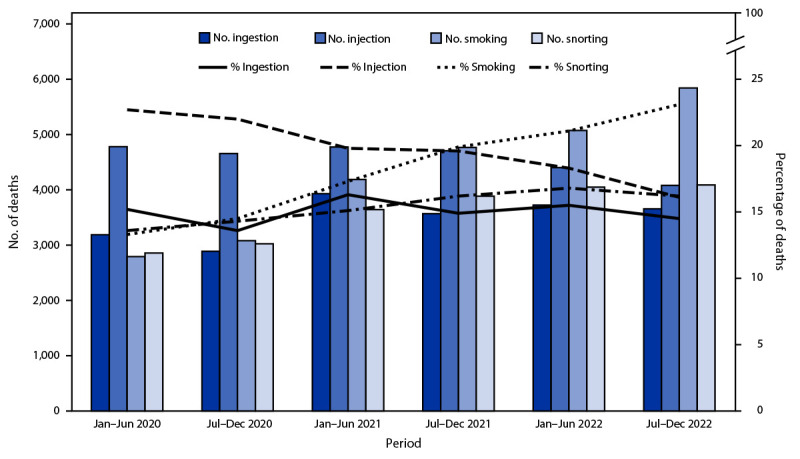
Number and percentage of drug overdose deaths with evidence of selected routes of drug use,^*^^,^^† ^by 6-month period of death (N = 139,740) — State Unintentional Drug Overdose Reporting System, 28 jurisdictions,^§^^,^^¶^ January 2020–December 2022 **Abbreviation:** SUDORS = State Unintentional Drug Overdose Reporting System. * Percentages with evidence of other routes (i.e., buccal, sublingual, suppository, or transdermal) (583; 0.4%) are not presented because of small sample sizes; decedents with drug use via these routes are included in the denominators. In addition, percentages of decedents with no information on route (68,260; 48.8%) are not shown; these decedents are also included in the denominators. ^†^ Routes of drug use are not mutually exclusive; decedents might have used multiple routes. ^§^ Alaska, Arizona, Colorado, Connecticut, Delaware, District of Columbia, Georgia, Illinois, Kansas, Kentucky, Maine, Maryland, Massachusetts, Minnesota, Nebraska, New Hampshire, New Jersey, North Carolina, Ohio, Oklahoma, Oregon, Pennsylvania, Rhode Island, Utah, Vermont, Virginia, Washington, and West Virginia. Illinois and Washington reported deaths from counties that accounted for ≥75% of drug overdose deaths in the respective state in 2017, per SUDORS funding requirements; all other jurisdictions reported deaths from the full jurisdiction. ^¶^ Jurisdictions were included if medical examiner or coroner reports and toxicology reports were available for ≥75% of deaths during January 2020–December 2022. Analysis was restricted to deaths with an available medical examiner or coroner report (139,740; 95.8% of all deaths).

During January 2020–December 2022, a total of 139,740 overdose deaths occurred in 28 jurisdictions; deaths increased 20.2%, from 21,046 during January–June 2020 to 25,301 during July–December 2022. The percentage of deaths with IMFs detected increased 8.4% from 71.4% during January–June 2020 to 77.4% during July–December 2022. Evidence of at least one route of drug use was documented in 71,480 (51.2%) overdose deaths. From January–June 2020 to July–December 2022, the number and percentage of overdose deaths with evidence of smoking increased 109.1% (from 2,794 to 5,843) and 73.7% (from 13.3% to 23.1%), respectively (Figure 1). The number and percentage of deaths with evidence of snorting increased 43.1% (from 2,858 to 4,090) and 19.1% (from 13.6% to 16.2%), respectively. In contrast, the number and percentage of overdose deaths with evidence of injection decreased 14.6% (from 4,780 to 4,080) and 29.1% (from 22.7% to 16.1%), respectively, from January–June 2020 to July–December 2022. Although the number of deaths with evidence of ingestion increased 14.6%, from 3,189 to 3,656, the percentage of such deaths declined 4.6%, from 15.2% to 14.5%.

The leading route of use in drug overdose deaths changed from injection during January–June 2020 (22.7% of deaths) compared with ingestion (15.2%), snorting (13.6%), and smoking (13.3%) to smoking during July–December 2022 (23.1% of deaths) compared with snorting (16.2%), injection (16.1%), and ingestion (14.5%). During July–December 2022, most deaths with evidence of smoking (79.7%), snorting (84.5%), or ingestion (86.5%) had no evidence of injection; among deaths with information on route of use, 81.9% had evidence of a noninjection route.

### Regional Trends

**FIGURE 2:**
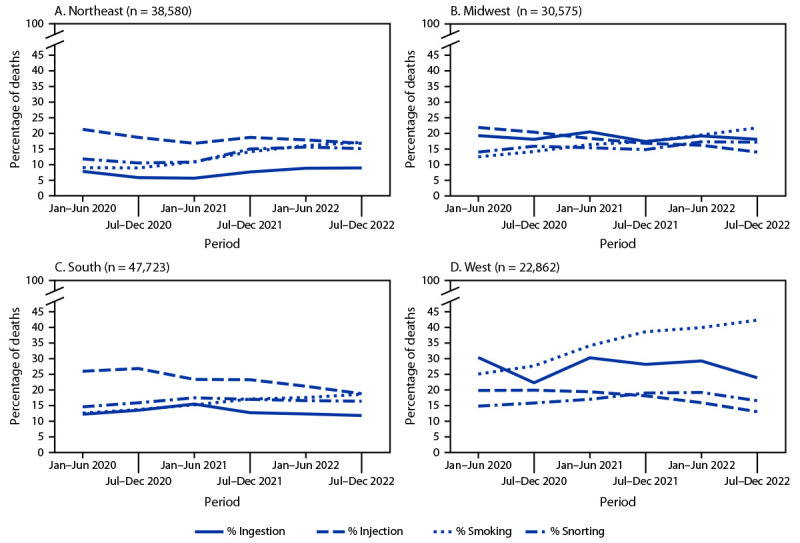
Percentage of drug overdose deaths with evidence of selected routes of drug use,* by U.S. Census Bureau region^†^ and 6-month period of death (N = 139,740) — State Unintentional Drug Overdose Reporting System, 28 jurisdictions,^§^ January 2020–December 2022 * Percentages with evidence of other routes (i.e., buccal, sublingual, suppository, or transdermal) are not presented because of small sample sizes (Panel A [Northeast]: 136, 0.4%; Panel B [Midwest]: 121, 0.4%; Panel C [South]: 223, 0.5%; and Panel D [West]: 103, 0.5%); decedents with drug use via these routes are included in the denominators. In addition, percentages of decedents with no information on route are not shown (Panel A: 22,541, 58.4%; Panel B: 15,381, 50.3%; Panel C: 22,571, 47.3%; and Panel D: 7,767, 34.0%); these decedents are also included in the denominators. ^†^ Analysis included some, but not all, of the jurisdictions in each U.S. Census Bureau region. *Northeast:* Connecticut, Maine, Massachusetts, New Hampshire, New Jersey, Pennsylvania, Rhode Island, and Vermont; *Midwest:* Illinois, Kansas, Minnesota, Nebraska, and Ohio; *South:* Delaware, District of Columbia, Georgia, Kentucky, Maryland, North Carolina, Oklahoma, Virginia, and West Virginia; *West:* Alaska, Arizona, Colorado, Oregon, Utah, and Washington. ^§^ Jurisdictions were included if medical examiner or coroner reports and toxicology reports were available for ≥75% of deaths during January 2020–December 2022. Analysis was restricted to deaths with an available medical examiner or coroner report (139,740; 95.8% of all deaths).

Regional trends were largely consistent with overall trends. The percentage of overdose deaths with evidence of smoking increased in all U.S. Census Bureau regions (Northeast: 91.0% increase, from 8.9% to 17.0%; Midwest: 75.0%, from 12.4% to 21.7%; South: 48.0%, from 12.5% to 18.5%; and West: 68.9%, from 25.1% to 42.4%) (Figure 2). The percentage of deaths with evidence of snorting increased in three regions (Northeast: 28.2%, from 11.7% to 15.0%; Midwest: 23.0%, from 13.9% to 17.1%; and South: 12.4%, from 14.5% to 16.3%). The percentage with evidence of injection decreased in all regions (Northeast: −21.2%, from 21.2% to 16.7%; Midwest: −36.2%, from 21.8% to 13.9%; South: −27.8%, from 25.9% to 18.7%; and West: −34.3%, from 19.8% to 13.0%). By July–December 2022, smoking was the most commonly identified route of use in overdose deaths in the Midwest (21.7%) and West (42.4%); injection and smoking were most common in the Northeast (16.7% and 17.0%, respectively) and South (18.7% and 18.5%, respectively).

### Trends by Drugs Detected

**FIGURE 3:**
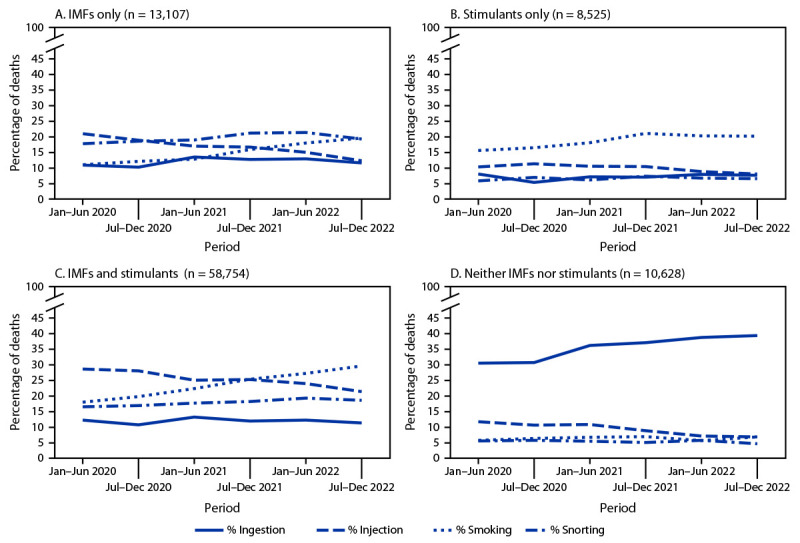
Percentage of drug overdose deaths with evidence of selected routes of drug use,* by drugs detected^†^^,^^§^^,^^¶^^,^**^,^^††^ and 6-month period of death — State Unintentional Drug Overdose Reporting System, 28 jurisdictions,^§§^ January 2020–December 2022 **Abbreviations:** IMFs = illegally manufactured fentanyls; SUDORS = State Unintentional Drug Overdose Reporting System. * Percentages with evidence of other routes (i.e., buccal, sublingual, suppository, or transdermal) are not presented because of small sample sizes (Panel A [IMFs only]: 23, 0.2%; Panel B [Stimulants only]: 11, 0.1%; Panel C [IMFs and stimulants]: 146, 0.2%; and Panel D [Neither IMFs nor stimulants]: 158, 1.5%); decedents with drug use via these routes are included in the denominators. In addition, percentages of decedents with no information on route are not shown (Panel A: 6,802, 51.9%; Panel B: 5,652, 66.3%; Panel C: 25,597, 43.6%; and Panel D: 5,435, 51.1%); these decedents are also included in the denominators. ^†^ Data on drugs detected come from postmortem toxicology reports; among decedents with a medical examiner or coroner report, analysis was further restricted to decedents with a toxicology report (136,466; 97.7% of decedents with a medical examiner or coroner report). ^§^ Ethanol and other selected drugs (e.g., naloxone and cotinine) were not considered a drug for this analysis; deaths categorized as IMFs only (Panel A) or stimulant only (Panel B) might have also had ethanol or these other selected drugs detected. ^¶^ Deaths with IMFs and stimulants detected (Panel C) could also have other drugs detected (e.g., prescription opioids). ** Deaths with neither IMFs nor stimulants detected primarily had prescription opioids (65.3%) or benzodiazepines (37.3%) detected. ^††^ Drug categories are not comprehensive; some deaths are excluded because they contain drug combinations that are not presented in the panels (e.g., deaths with only IMFs and prescription opioids detected). ^§§^ Alaska, Arizona, Colorado, Connecticut, Delaware, District of Columbia, Georgia, Illinois, Kansas, Kentucky, Maine, Maryland, Massachusetts, Minnesota, Nebraska, New Hampshire, New Jersey, North Carolina, Ohio, Oklahoma, Oregon, Pennsylvania, Rhode Island, Utah, Vermont, Virginia, Washington, and West Virginia. Illinois and Washington reported deaths from counties that accounted for ≥75% of drug overdose deaths in the respective state in 2017, per SUDORS funding requirements; all other jurisdictions reported deaths from the full jurisdiction.

Among overdose deaths with only IMFs detected (13,107; 9.6%), deaths with both IMFs and stimulants detected (58,754; 43.1%), and deaths with only stimulants detected (8,525; 6.2%), the percentage with evidence of smoking increased, and the percentage with evidence of injection decreased from January–June 2020 to July–December 2022 (Figure 3). For IMFs only, the percentage of overdose deaths with evidence of smoking increased 78.9%, from 10.9% to 19.5%, whereas the percentage with evidence of injection decreased 41.6%, from 20.9% to 12.2%. Among deaths with both IMFs and stimulants detected, the percentage with evidence of smoking increased 65.4%, from 17.9% to 29.6%, whereas the percentage with evidence of injection decreased 25.5%, from 28.6% to 21.3%. A similar pattern was observed among deaths with only stimulants detected (smoking: 29.7% increase, from 15.5% to 20.1%; injection: 22.5% decrease, from 10.2% to 7.9%). Among deaths with neither IMFs nor stimulants detected (10,628; 7.8%), the percentage with evidence of smoking did not change, and the percentage with evidence of injection decreased 42.2% (11.6% to 6.7%); ingestion was the most common route during July–December 2022 (39.4% of deaths) and throughout the study period.

## Discussion

The percentage of drug overdose deaths with evidence of smoking increased sharply in all U.S. regions from 2020 to 2022, indicating the importance of an updated response. By late 2022, among decedents with information on route of drug use, more than three fourths had evidence of a noninjection route, highlighting the diversification of methods through which they used drugs.

From January–June 2020 to July–December 2022, the number of overdose deaths with evidence of smoking doubled, and the percentage of deaths with evidence of smoking increased across all geographic regions. By late 2022, smoking was the predominant route of use among drug overdose deaths overall and in the Midwest and West regions. Increases were most pronounced when IMFs were detected, with or without stimulants. Increases in the number and percentage of deaths with evidence of smoking, and the corresponding decrease in those with evidence of injection, might be partially driven by 1) the transition from injecting heroin to smoking IMFs (*3*,*4*), 2) increases in deaths co-involving IMFs and stimulants that might be smoked^††††^ (*1*), and 3) increases in the use of counterfeit pills, which frequently contain IMFs and are often smoked (*7*). Motivations for transitioning from injection to smoking include fewer adverse health effects (e.g., fewer abscesses), reduced cost and stigma, sense of more control over drug quantity consumed per use (e.g., smoking small amounts during a period versus a single injection bolus), and a perception of reduced overdose risk among persons who use drugs (*3*,*5*,*8*). These motivations might also signify lower barriers for initiating drug use by smoking, or for transitioning from ingestion to smoking; compared with ingestion, smoking can intensify drug effects and increase overdose risk (*9*). Despite some risk reduction associated with smoking compared with injection (e.g., fewer bloodborne infections), smoking carries substantial overdose risk because of rapid drug absorption (*5*,*9*).

Nearly 80% of overdose deaths with evidence of smoking had no evidence of injection; persons who use drugs by smoking but do not inject drugs might not use traditional syringe services programs where harm reduction messaging and supplies are often provided. In response, some jurisdictions have adapted harm reduction services to provide safer smoking supplies or established health hubs to expand reach to persons using drugs through noninjection routes.^§§§§^ In addition, harm reduction services (e.g., peer outreach and provision of fentanyl test strips for testing drug products and naloxone to reverse opioid overdoses), messaging specific to smoking drugs, and linkage to treatment for substance use disorders can be integrated into other health care delivery (e.g., emergency departments) and public safety (e.g., drug diversion) settings.

The percentage and number of deaths with evidence of injection decreased across regions and drug categories. Observed decreases might reflect transitions to noninjection routes and response to public health efforts to reduce injection drug use because of its risk for overdose and infectious disease transmission (*3*,*4*,*10*). Despite these declines, more than 4,000 drug overdose deaths had evidence of injection during July–December 2022. Syringe services programs help to engage persons who use drugs in services (*10*); sustained efforts to provide sterile injection supplies, additional harm reduction tools, and linkage to treatment for substance use disorders, including medications for opioid use disorder, are important for further reduction in the number of overdose deaths from injection drug use. Lessons learned from implementing syringe services programs could be applied to other harm reduction and outreach models to reach more persons who use drugs by any route.

### Limitations

The findings in this report are subject to at least four limitations. First, analyses included 28 jurisdictions; results might not be generalizable to the rest of the United States. Second, for nearly one half of deaths, no information about route of drug use was available; thus, percentages of deaths with evidence of each route are underestimated. However, no notable differences by time or demographic characteristics among deaths with and without route of drug use information were identified. Third, percentages of noninjection routes are likely underestimated more than those with injection because evidence of injection is easier to identify (e.g., syringes) than evidence of other routes (e.g., stems and straws can be evidence of snorting or smoking). Finally, routes could not be linked to the use of a specific drug unless only one drug class was detected. Analyses of single drug classes detected (IMFs only and stimulants only) were presented to better link routes to drugs.

### Implications for Public Health Practice

Routes of drug use have implications for overdose risk, infectious disease transmission, and harm reduction services (*5*). Although unsafe injection drug use practices might be most risky in terms of infectious disease transmission, other routes, particularly smoking, still carry substantial overdose risk (*9*). Sharp increases in deaths with evidence of smoking and continued prevalence of other routes of drug use highlight the importance of 1) expanded messaging emphasizing overdose risk associated with smoking and other routes; 2) continued and expanded support for syringe services programs to provide comprehensive, integrated health services; and 3) enhanced outreach and harm reduction services (e.g., peer outreach and provision of fentanyl test strips and naloxone) across multiple settings for persons using drugs by smoking and other routes. These strategies might increase access to lifesaving services for persons who use drugs through all routes.

## Footnotes

* https://www.cdc.gov/nchs/nvss/vsrr/drug-overdose-data.htm (Accessed January 11, 2024).

†
https://adai.uw.edu/wordpress/wp-content/uploads/dlm_uploads/FentanylQualitativeReport2023.pdf

§
https://www.cdc.gov/drugoverdose/fatal/sudors.html

¶ Evidence of ingestion included witness reports of taking pills or tablets orally or ingesting liquid orally (e.g., liquid methadone), or the discovery of prescription pills, prescription bottles, liquid substances, or vials for containing liquid substances at the scene of the overdose or on the decedent’s body.

** Evidence of injection included witness reports of injecting drugs, items used to prepare and inject substances found at the scene (e.g., needles, cookers, filters, tourniquets, or alcohol pads), or track marks found on the decedent that appeared to be recent.

†† Evidence of smoking included witness reports of smoking drugs or drug paraphernalia at the overdose scene associated with smoking (e.g., pipes, stems, aluminum foil, vape pens, matches, disposable lighters, or gas torches). Fewer than 6.0% of deaths with evidence of smoking had vape pens or e-cigarettes endorsed as evidence; fewer than 3.0% had vape pens or e-cigarettes endorsed with no other evidence of smoking.

§§ Evidence of snorting included witness reports of snorting drugs, drug paraphernalia at the overdose scene associated with snorting (e.g., razor blades or credit cards used to chop and separate powder; straws, rolled paper, dollar bills, or tubes for nasal inhalation; or powder visible on a table or mirror), or powder on the decedent’s nose.

¶¶ Alaska, Arizona, Colorado, Connecticut, Delaware, District of Columbia, Georgia, Illinois, Kansas, Kentucky, Maine, Maryland, Massachusetts, Minnesota, Nebraska, New Hampshire, New Jersey, North Carolina, Ohio, Oklahoma, Oregon, Pennsylvania, Rhode Island, Utah, Vermont, Virginia, Washington, and West Virginia. Illinois and Washington reported deaths from counties that accounted for ≥75% of drug overdose deaths in the respective state in 2017, per SUDORS funding requirements; all other jurisdictions reported deaths from the full jurisdiction.

*** Jurisdictions were included if medical examiner or coroner reports and toxicology reports were available for ≥75% of deaths during January 2020–December 2022. Analyses were restricted to decedents with an available medical examiner or coroner report (139,740; 95.8% of all deaths).

††† U.S. Census Bureau regions were used to stratify jurisdictions into geographic regions (https://www2.census.gov/geo/pdfs/maps-data/maps/reference/us_regdiv.pdf). Region analysis included eight of nine jurisdictions in the Northeast Region, five of 12 jurisdictions in the Midwest Region, nine of 17 jurisdictions in the South Region, and six of 13 jurisdictions in the West Region.

§§§ Fentanyl was classified as likely illegally manufactured using toxicology, scene, and witness evidence. For the 8.1% of deaths involving fentanyl that had insufficient evidence for classification as illegal or prescription, fentanyl was classified as illegal because the majority of fentanyl overdose deaths involve illegal fentanyl. All fentanyl analogs except alfentanil, remifentanil, and sufentanil, which have legitimate human medical use, were included as IMFs.

¶¶¶ Analysis of drugs detected was restricted to decedents with an available toxicology report (136,466; 97.7% of deaths with a medical examiner or coroner report).

**** 45 C.F.R. part 46.102(l)(2), 21 C.F.R. part 56; 42 U.S.C. Sect. 241(d); 5 U.S.C. Sect. 552a; 44 U.S.C. Sect. 3501 et seq.

††††
https://adai.uw.edu/wordpress/wp-content/uploads/SaferSmokingBrief_2022.pdf

§§§§ https://www.maricopa.gov/DocumentCenter/View/86245/OUD-SUD-Needs-Assessment-Final-Report?bidId=; https://www.cdph.ca.gov/Programs/CID/DOA/CDPH%20Document%20Library/HR_Supplies_Clearinghouse_Factsheet_FINAL.pdf; https://www.health.ny.gov/diseases/aids/consumers/prevention/
